# Investigating the Genetic Links Between Immune Cell Profiles and Bladder Cancer: A Multidisciplinary Bioinformatics Approach

**DOI:** 10.3390/biomedicines13051203

**Published:** 2025-05-15

**Authors:** Jin Zhang, Zhongji Jiang, Jiali Jin, Gaohaer Kadeerhan, Hong Guo, Dongwen Wang

**Affiliations:** 1 Department of the First Clinical Medical College, Shanxi Medical University, Taiyuan 030001, China; 2 Department of Urology, National Cancer Center/National Clinical Research Center for Cancer/Cancer Hospital & Shenzhen Hospital, Chinese Academy of Medical Sciences and Peking Union Medical College, Shenzhen 518116, China; 3 School of Medicine, Southern University of Science and Technology, Shenzhen 518055, China; 4 Central Laboratory, National Cancer Center/National Clinical Research Center for Cancer/Cancer Hospital & Shenzhen Hospital, Chinese Academy of Medical Sciences and Peking Union Medical College, Shenzhen 518116, China; 5 Department of Urology, First Hospital of Shanxi Medical University, Taiyuan 030001, China

**Keywords:** bladder cancer, immune microenvironment, mendelian randomization, machine learning, diagnostic biomarkers

## Abstract

**Background**: Bladder cancer (BC) is a common malignancy in the urinary system, with an increasing incidence rate. Immune cell infiltration within the tumor microenvironment (TME) plays a crucial role in BC progression and treatment response. However, the immune cell composition of the TME presents a significant challenge to the effectiveness of current therapeutic strategies. **Methods**: We performed bidirectional Mendelian randomization (MR) analysis to investigate the impact of immune cells on BC risk. Single nucleotide polymorphisms (SNPs) related to immune cells were annotated, and candidate genes associated with BC risk were identified. Differential expression analysis identified immune-related differentially expressed genes (iDEGs), and a protein–protein interaction (PPI) network along with functional enrichment analysis were conducted to explore their roles in tumor development. Machine learning-based feature selection was applied to identify potential biomarkers and therapeutic targets. **Results**: MR analysis revealed eight immune cell subtypes significantly associated with BC. Using SNPs linked to these immune cells, 129 candidate genes were identified through the SNPense tool and cross-referenced with differentially expressed genes in BC, resulting in identification of 28 iDEGs. Machine learning identified five potential diagnostic biomarkers (*COLEC12*, *TMCC1*, *CEP55*, *KLK3*, *COL4A1*) with an AUC of 0.903, which are implicated in immune modulation and cancer progression. **Conclusions**: This study provides new insights into immune mechanisms in BC and identifies promising biomarkers for early diagnosis and therapeutic intervention.

## 1. Introduction

Bladder cancer (BC) ranks as one of the most common malignancies in the urinary system, second only to prostate cancer. In recent years, there has been a noticeable upward trend in its age-standardized incidence rate [1,2]. BC is clinically classified into two categories: non-muscle-invasive (NMIBC) and muscle-invasive (MIBC). While NMIBC represents about 75–85% of all BC cases, it is notably associated with a high recurrence rate [3]. Following transurethral resection of bladder tumors (TURBT), the routine use of intravesical chemotherapy and Bacillus Calmette–Guérin (BCG) instillation plays a critical role in minimizing the likelihood of tumor recurrence [4,5].

BC is widely recognized as an immunogenic tumor, with studies demonstrating the abundant expression of tumor-associated antigens and tumor-specific antigens by BC cells [6,7,8]. Immune cell infiltration in the tumor microenvironment (TME) of BC plays a critical role in the progression of the disease. This infiltration has been shown to not only influence BC progression but also serves as an important predictor for the effectiveness of perioperative chemotherapy and immunotherapy [9,10,11]. Moreover, the immune cell composition of the TME reflects a major challenge to the effectiveness of current therapeutic strategies, as immune checkpoint inhibitors (ICIs) show efficacy only in a limited patient population, primarily due to the immunosuppressive nature of the TME [12,13,14]. The TME in BC contains various immune cells, including T cells, B cells, NK cells, and regulatory T cells (Tregs), each playing distinct roles in tumor progression and immune evasion [15,16,17,18,19,20]. CD8+ and CD4+ T cells are crucial for tumor elimination, but their function is often suppressed within the TME [16,17]. B cells influence immunity through antigen presentation and cytokine secretion [21]. NK cells are vital for early tumor defense, directly killing tumor cells without prior sensitization [19]. Tregs, which maintain immune tolerance, often inhibit anti-tumor responses, allowing tumor cells to evade immune surveillance [20]. The balance and function of these immune cells significantly affect tumor progression and response to immunotherapy, highlighting the complexity of the TME and the need for targeted strategies. Therefore, identifying immune cells involved in the development of BC, along with their associated genes, may offer novel therapeutic avenues for improving BC treatment strategies.

Mendelian randomization (MR) is a powerful epidemiological tool that utilizes genetic variants as instrumental variables (IVs) [22,23,24]. MR allows researchers to establish causal links between risk factors and disease progression by simulating randomized controlled trials, thereby providing essential insights into the underlying factors that contribute to disease progression. By utilizing IVs that are randomly distributed at conception, MR minimizes confounding and mitigates reverse causation, providing reliable insights into causal pathways. Data from transcriptomic analyses, obtained through The Cancer Genome Atlas (TCGA) and the Gene Expression Omnibus (GEO) database, provide comprehensive gene expression profiles. These resources are crucial in advancing our comprehension of the mechanisms that drive BC and in the formulation of targeted therapeutic strategies.

For this investigation, we systematically evaluated the potential causal links between immune cell populations and BC using bidirectional MR analysis. Additionally, by annotating SNPs related to immune cell genetics, we uncovered a set of candidate genes with potential causal relevance to BC susceptibility. Transcriptomic data were integrated to pinpoint immune-related differentially expressed genes (iDEGs), further enhancing the understanding of immune mechanisms. These genes were analyzed through the construction of a protein–protein interaction (PPI) network and functional enrichment approaches to investigate their potential biological roles in tumor development. Subsequently, five hub genes were identified through machine learning-based feature selection. In conclusion, our study offers valuable insights into the development of treatment strategies for BC and identifies more effective diagnostic biomarkers for the disease. These findings lay the foundation for enhanced diagnosis and treatment of BC.

## 2. Materials and Methods

### 2.1. Data Sources

Data summary for immune-related traits, used as exposure information, were retrieved from the GWAS Catalog (GCST90001391 to GCST90002121), with data available as of 1 July 2024 [25]. This study examined 731 immune phenotypes through flow cytometry on peripheral blood samples obtained from 3757 Sardinian individuals. The analysis included 118 absolute cell counts (AC), 389 median fluorescence intensities (MFI) of surface antigens, 32 morphological parameters (MP), and 192 relative cell counts (RC). These four categories of metrics were used to characterize and assess immune cell populations quantitatively. Furthermore, approximately 22 million SNPs were identified in the analysis.

To conduct the analysis, outcome data were retrieved from the FinnGen R11 release, a comprehensive biobank-driven genomic resource https://www.finngen.fi/en (accessed on 1 July 2024) [26]. The diagnostic criteria for BC were established based on ICD codes: ICD-10 (C67), ICD-9 (188), and ICD-8 (188). This dataset included 2574 cases and 345,118 controls, with all controls confirmed to have no history of any cancer diagnosis.

For the transcriptomic data, we applied rigorous inclusion criteria to ensure both its quality and relevance. The criteria included the following: (1) data type: expression profiling using arrays, real-time polymerase chain reaction (RT-PCR), and next-generation sequencing; (2) species: Homo sapiens; (3) disease: confirmed bladder cancer in the original study; (4) sample source: bladder tissue; (5) sample content: RNA expression data from both control and BC groups, without any intervention. As of 1 July 2024, we retrieved several BC-related datasets (TCGA-BLCA [27], GSE3167 [28], and GSE13507 [29]) from publicly available databases. These datasets include samples from both control and BC groups. Due to the limited availability of control samples, additional normal bladder tissue data were sourced from the GTEx database [30] also on 1 July 2024. The TPM obtained from TCGA were transformed using log2 (TPM + 1) to normalize the data and mitigate the right-skewed distribution commonly observed in RNA sequencing datasets.

### 2.2. Batch Effect Removal

To mitigate batch effects across the four datasets (TCGA-BLCA, GSE3167, GSE13507, and GTEx-Bladder), the “ComBat” function provided by the “sva” package (v3.5.0) [31]. The effectiveness of correction was evaluated via principal component analysis (PCA), allowing comparison of data structure before and after batch adjustment.

### 2.3. Mendelian Randomization Analysis

To explore the potential causal effects of immune system variation on BC susceptibility, we implemented two-sample MR analyses involving 731 immune cell phenotypes, utilizing the TwoSampleMR package (v0.5.11) [32]. The main analysis utilized the random effects inverse variance-weighted (IVW) approach. To evaluate the robustness of the MR results, multiple statistical tests were applied. Heterogeneity was assessed using a combination of Cochran’s Q test and MR-PRESSO global test, with a significance threshold set at *p* < 0.05. MR-PRESSO not only accounts for horizontal pleiotropy but also identifies and removes outlier SNPs, whereas MR-Egger focuses on detecting pleiotropy through intercept testing [33]. Horizontal pleiotropy was examined through the MR-Egger intercept, with statistical significance defined as a *p*-value below 0.05 indicating its presence. Additionally, any analyses involving fewer than three SNPs were excluded from consideration.

For MR to yield valid causal inferences, three essential assumptions must be met: (1) genetic variants must have a strong relevance to the exposure, (2) they should not be associated with confounders, and (3) their effect on the outcome must occur exclusively through the exposure pathway [34].

### 2.4. Reverse Mendelian Randomization Analysis

A reverse MR analysis was performed to assess whether BC exerts a causal effect on immune cells, treating BC as the exposure and immune cells as the outcome. The methodology for this analysis was consistent with that used in the forward MR analysis to maintain consistency between both approaches. By comparing the results of both analyses, we aimed to gain a more nuanced understanding of the causal associations between BC and immune cell profiles.

### 2.5. Profiling of Differentially Expressed Genes

We began by mapping the corresponding SNPs to genes using the SNPense tool https://biit.cs.ut.ee/gprofiler/snpense (accessed on 2 November 2024), which links human SNP rsIDs to gene names [35]. SNPense is designed to prioritize genetic variants mapped to one or more protein-coding genes annotated in the Ensembl database, with all relevant data retrieved extracted from the Ensembl Variation resource. Following this, we extracted immune-related gene expression data from both the control and BC groups within the merged datasets to conduct differential expression analysis. Genes with a significant positive association in MR analysis (*p* < 0.05) and differential expression in BC were classified as immune-related differentially expressed genes (iDEGs). The results were visualized in a box plot. Using the “circlize” package (v0.4.15), we mapped the positions of iDEGs across chromosomes, with their distribution displayed in a circos plot.

### 2.6. Immune Cells Infiltration

Immune cell infiltration in BC was assessed using the CIBERSORT algorithm, with 1000 permutations applied to estimate the relative proportions of 22 distinct immune cell populations [36]. The association between iDEGs and immune infiltration levels was subsequently examined, and the findings were presented using bar charts. Simultaneously, single-sample gene set enrichment analysis (ssGSEA) was used to quantify and contrast immune landscape profiles between tumor and control tissues. Box plots were constructed to visualize the results, highlighting the differences in immune cell composition. To further investigate the interplay between gene expression and immune infiltration, correlation analyses were performed by aligning iDEG expression levels with ssGSEA-derived scores, and the computed correlation coefficients were displayed in a heatmap for comprehensive visualization.

### 2.7. GO/KEGG Enrichment Analysis

Functional annotation and pathway enrichment of iDEGs were conducted with the clusterProfiler R package (v4.8.3) [37], aiming to uncover relevant biological processes and underlying mechanisms of disease. The Gene Ontology (GO) framework was employed to organize gene annotations into the three principal GO domains: biological process (BP), molecular function (MF), and cellular component (CC), each providing insights into different aspects of gene function. Kyoto Encyclopedia of Genes and Genomes (KEGG) pathway analysis complemented the GO results by identifying enriched metabolic and signaling pathways associated with iDEGs. A significance level of *p* < 0.05 was set to assess the relevance of enrichment in all analyses.

### 2.8. Construction of the Protein–Protein Interaction Network

To investigate the protein–protein interactions related to disease mechanisms, we constructed an interaction network with the STRING database https://cn.string-db.org/ (accessed on 2 April 2025), setting a minimum confidence score threshold of 0.15 to filter out weak associations [38]. The network was then imported into Cytoscape (version 3.9.1) [39] for visualization and further topological exploration. Default settings were retained for all other parameters to maintain consistency across analyses. By examining the resulting network structure, we gained valuable insight into the functional relationships among proteins and their potential roles in the cellular environment.

### 2.9. Machine Learning Algorithms

Using the expression profiles of iDEGs, nine machine learning models were established. Prediction functions were computed for each model, and the most effective algorithm was identified based on predictive performance. Residual box plots and receiver operating characteristic (ROC) curve analyses were performed to identify key features, which facilitated the selection of representative genes. These genes were subsequently used to construct a nomogram that integrated their expression levels in both BC and control tissue samples. To comprehensively assess the nomogram’s effectiveness, its predictive accuracy and capacity for generalization were examined through calibration curves and decision curve analysis. Validation was performed with an independent dataset to replicate the model and evaluate its stability, with the ROC curve illustrating classification performance.

### 2.10. Statistical Analysis

All statistical analyses were performed in R (version 4.2.1). For comparisons between two independent groups, Student’s *t*-test was employed, whereas the Wilcoxon signed-rank test was used for analyzing paired data. For comparisons involving three or more groups, one-way ANOVA and the Kruskal–Wallis test were performed. To evaluate correlations between variables, the Spearman rank correlation test was utilized.

## 3. Results

### 3.1. Study Design

**Figure 1:**
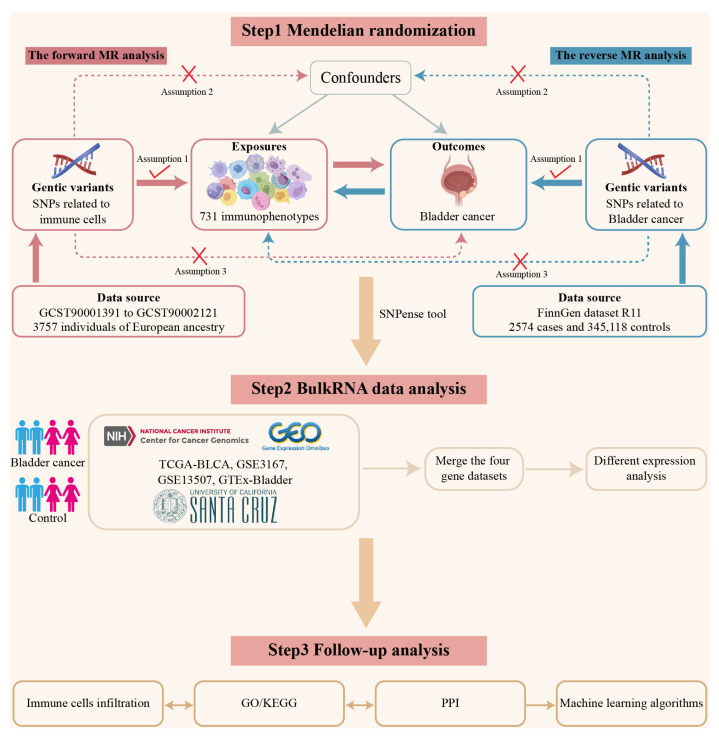
Overview of the analysis workflow. This flow plot summarizes the three-step process: (1) Bidirectional Mendelian randomization (MR) analysis to assess immune cells’ impact on bladder cancer (BC) risk. (2) Merging RNA data to identify immune-related differentially expressed genes (iDEGs). (3) Follow-up analyses, including functional enrichment, PPI network, and machine learning to identify biomarkers and therapeutic targets. In Step 1, red and blue arrows represent the forward and reverse MR analyses, respectively. Symbols: A check mark (√) indicates a relevant association; a cross (×) indicates no association. According to the core Mendelian randomization assumptions, the genetic variants (SNPs) should be strongly associated with the exposure (√), but not associated with the outcome or any confounders (×).

This study used a three-step approach to examine the impact of immune cells on BC risk. First, bidirectional MR analysis was conducted using SNPs associated with immune cells. In the forward MR analysis, immune cells were considered the exposure factor, with BC as the outcome. The data for this analysis were sourced from the GCST90001391 and GCST90002121 datasets, which included 3757 individuals of European ancestry. In the reverse MR analysis, BC risk was used as the exposure factor, with immune cell types as the outcome. SNPs associated with BC risk were retrieved from the FinnGen dataset (R11). In the second step, bulk expression data from TCGA-BLCA, GSE3167, GSE13507, and GTEx-Bladder were combined. The SNPense tool https://biit.cs.ut.ee/gprofiler/snpense (accessed on 2 November 2024) was used to map SNPs associated with these immune cells to identify candidate genes. Differential expression analysis was performed to identify iDEGs, which were cross-referenced with immune cell data and further examined for their potential roles in tumor progression. In the third step, follow-up analysis included immune cell infiltration analysis within the TME, functional enrichment through GO/KEGG, and PPI network analysis to explore the biological roles of iDEGs. Finally, machine learning-based feature selection was applied to identify potential biomarkers and therapeutic targets. The overall workflow is illustrated in Figure 1.

### 3.2. Estimating the Causal Impact of Immune Cells on Bladder Cancer

**Figure 2:**
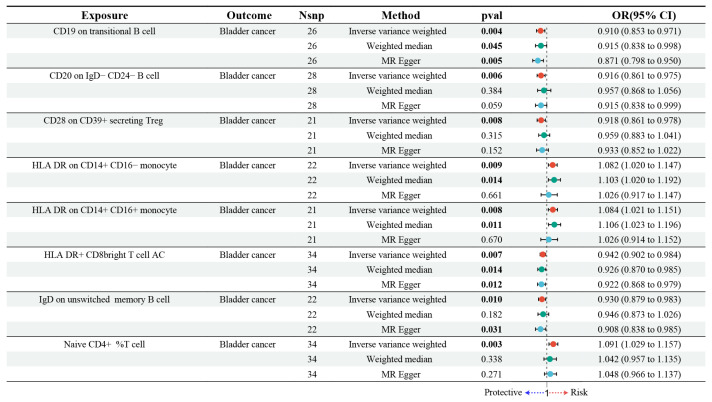
Forest plot depicting the findings from MR analysis exploring the relationship between immune cell types and BC. “Nsnp” represents the number of single nucleotide polymorphisms (SNPs); “OR” refers to the odds ratio; and “CI” denotes the confidence interval. “Naive CD4+ %T cell” indicates the proportion of naive CD4+ T cells among the total T cell population. “CD8 bright T cell AC” refers to CD8+ T cells with bright (high intensity) expression of the CD8 receptor, with “AC” representing absolute counts.

A total of 18,621 SNPs were selected as IVs based on a relaxed genome-wide significance threshold (*p* < 1 × 10^−5^) from GWAS data [40]. Linkage disequilibrium pruning was subsequently applied with an r^2^ threshold of less than 0.001 across a 10 Mb window. To verify the robustness of these instrumental variables, we calculated the F-statistic for each SNP. All SNPs demonstrated F-statistics greater than 10, which indicates a low risk of weak instrument bias (Table S1). Subsequent to the MR analysis (Table S2), three key filtering criteria were applied: consistency of effect direction, horizontal pleiotropy assessment, and heterogeneity evaluation. SNPs exhibiting inconsistent effect directions were excluded. Horizontal pleiotropy was assessed by applying the MR-Egger intercept test (Table S3) alongside MR-PRESSO analysis. Genes identified with significant pleiotropy (*p* < 0.05) were removed from the analysis for further evaluation. Heterogeneity was assessed through Cochran’s Q test, with detailed results provided in Table S4. Based on the analysis, we identified eight immune cell types potentially causally related to BC, as illustrated in Figure 2. Specifically, higher levels of CD19 on transitional B cells, CD20 on IgD− CD24− B cells, CD28 on CD39+ secreting Treg, HLA-DR+ CD8 bright AC, and IgD on unswitched memory B cells were associated with a decreased risk of BC. Conversely, increased levels of HLA-DR expression on CD14+ CD16− monocytes, HLA-DR on CD14+ CD16+ monocytes, and the percentage of Naive CD4+ T cells were found to correlate with a higher risk of BC.

### 3.3. Estimating the Causal Impact of Bladder Cancer on Immune Cells

**Figure 3:**
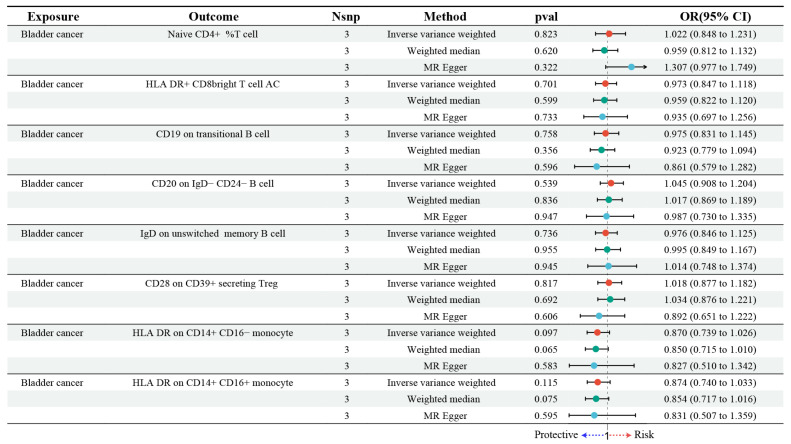
Forest plot illustrating the reverse MR analysis between BC and immune cells. “Nsnp” represents the number of SNPs; “OR” refers to the odds ratio; and “CI” denotes the confidence interval. “Naive CD4+ %T cell” indicates the proportion of naive CD4+ T cells among the total T cell population. “CD8 bright T cell AC” refers to CD8+ T cells with bright (high intensity) expression of the CD8 receptor, with “AC” representing absolute counts.

In this study, we employed genetic susceptibility to BC as the exposure to perform reverse MR analyses on eight immune cell types identified through MR models. The findings, presented in Figure 3 and detailed in Table S5, indicated that BC did not exert any significant causal influence on these immune cells. Specifically, the random effects model produced OR close to 1, with confidence intervals that included the null value, indicating no evidence of causal association. For instance, no significant associations were observed for Naive CD4+ %T cells (OR = 1.022, 95% CI: 0.848 to 1.231, *p* = 0.823) or HLA-DR+ CD8 bright T cell AC (OR = 0.973, 95% CI: 0.847 to 1.118, *p* = 0.701). The consistency of these findings was confirmed by complementary MR methods, such as the weighted median and MR-Egger approaches, which provided further evidence supporting the robustness of the results.

Our analysis indicates no evidence of reverse causality between BC and immune cell profiles, suggesting that BC does not affect the differentiation or activity of these immune cells. The lack of reverse causality supports the hypothesis that immune cells function primarily as upstream modulators, rather than serving as downstream targets of BC.

### 3.4. Identification of iDEGs in Bladder Cancer

**Figure 4:**
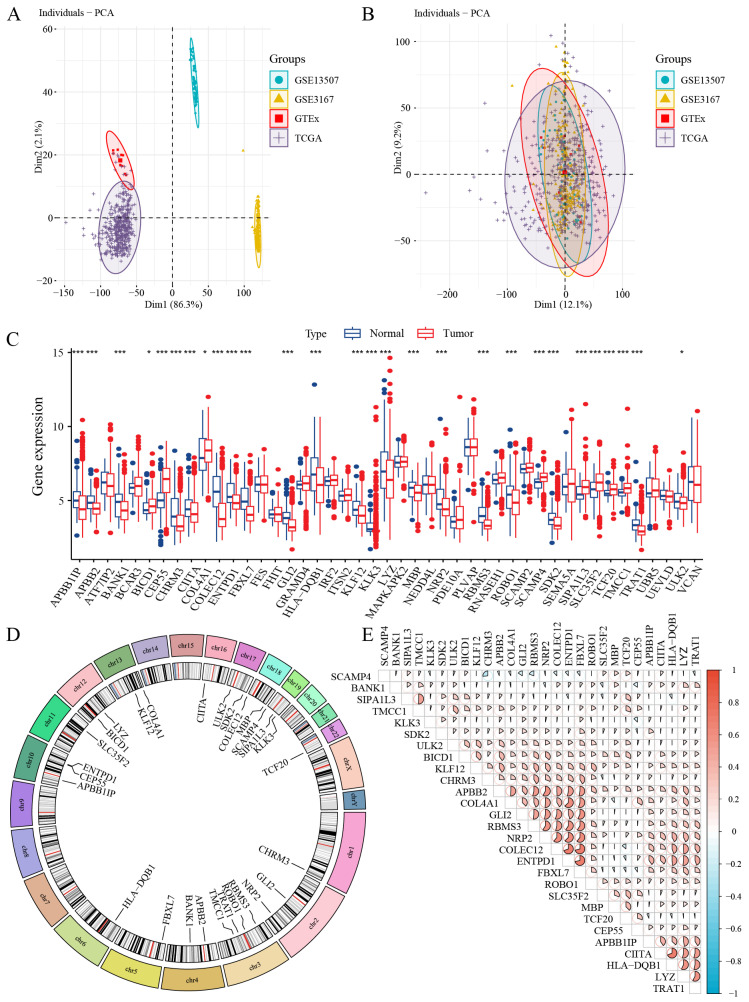
Integration of BC datasets and identification of iDEGs in BC. (**A**) PCA of the four original bladder cancer-related datasets before correcting for batch effects. (**B**) PCA after the integration of bladder cancer-related datasets, following batch effect adjustment. (**C**) Box plot illustrating the differential expression analysis of iDEGs across the control and BC cohorts. (**D**) Circular plot highlighting the chromosomal locations of iDEGs. (**E**) Heatmap visualizing the correlation analysis of iDEGs. * *p* < 0.05, *** *p* < 0.001.

We obtained the BC transcriptome data from TCGA, GEO, and GTEx (Table S6). After performing batch effect correction, integration, and normalization, we analyzed a total of 646 BC samples and 110 control samples. The processing workflow is summarized in Figure 4A,B, which illustrate a significant reduction in batch effects post-correction.

Using SNPs associated with significantly identified immune cells through SNPense tool, we mapped these SNPs to 129 gene names (Table S7). A total of 28 genes exhibiting significant differential expression were classified as immune-related differentially expressed genes (iDEGs). These genes are depicted in Figure 4C and Table S8. Among these, *BICD1*, *CEP55*, *COL4A1*, *SCAMP4*, *SIPA1L3*, *SLC35F2*, *TCF20*, and *TMCC1* were upregulated in tumor samples, while *APBB1IP*, *APBB2*, *BANK1*, *CHRM3*, *CIITA*, *COLEC12*, *ENTPD1*, *FBXL7*, *GLI2*, *HLA-DQB1*, *KLF12*, *KLK3*, *LYZ*, *MBP*, *NRP2*, *RBMS3*, *ROBO1*, *SDK2*, *TRAT1*, and *ULK2* were downregulated. The chromosomal locations of these significantly differentially expressed iDEGs are displayed in Figure 4D. Further, correlation analysis within the BC samples revealed predominantly positive correlations among the iDEGs, as shown in Figure 4E.

### 3.5. Evaluation of Immune Infiltration Patterns

**Figure 5:**
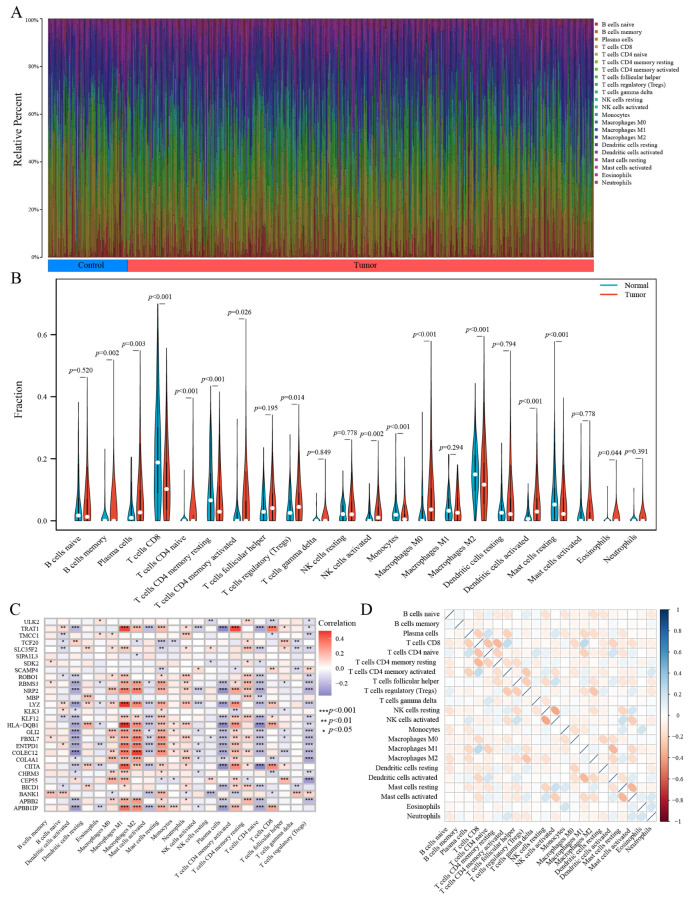
Comprehensive assessment of immune cell infiltration in BC. (**A**) Stacked bar chart illustrating the distribution of immune cell populations in BC and control samples. (**B**) Violin plot comparing the distribution and relative abundance of 22 immune cell subsets between control and BC groups. (**C**) Heatmap depicting the correlations between iDEGs and the 22 immune cell types. (**D**) Heatmap showing the intercorrelations among the 22 immune cell types. * *p* < 0.05, ** *p* < 0.01, and *** *p* < 0.001.

The iDEGs identified in this study may be integral in modulating immune responses and influencing the progression of BC. To further explore the connection between these genes and immune cells, the CIBERSORT algorithm was utilized to infer the characteristics of immune cells and their association with iDEGs in BC. The expression levels of 22 distinct immune cell types across the combined samples are presented in Figure 5A and detailed in Table S9. Notable differences in the proportions of specific immune cell subtypes were observed between BC cases and controls, as shown in Figure 5B. Particularly, a significant downregulation of T cells CD8, resting memory T cells CD4, monocytes, M2 macrophages, and resting mast cells was observed in BC samples. Conversely, memory B cells, plasma cells, naive CD4+ T cells, activated memory CD4+ T cells, regulatory T cells (Tregs), activated NK cells, M0 macrophages, activated dendritic cells, and eosinophils exhibited significant upregulation in the tumor samples.

Moreover, correlation analysis between iDEGs and these 22 immune cell types revealed significant associations, as shown in Figure 5C. HLA-DQB1, for instance, was significantly negatively correlated with activated dendritic cells, eosinophils, plasma cells, naive CD4+ T cells, and regulatory T cells (Tregs). Conversely, it was positively correlated with resting dendritic cells, M1 macrophages, M2 macrophages, resting mast cells, monocytes, neutrophils, activated memory CD4+ T cells, and CD8+ T cells. These results suggest a complex interaction between iDEGs and immune cell infiltration, which may influence the immune microenvironment’s role in BC development.

In order to investigate potential interactions among immune cells within the TME, we conducted a Spearman correlation analysis based on the relative abundances of 22 immune cell types, which were estimated using the CIBERSORT algorithm. The correlation coefficients revealed distinct relationships between immune cell populations. For example, M2 macrophages were positively correlated with Tregs, suggesting a possible cooperative role in promoting an immunosuppressive network. Additionally, CD8+ T cells showed a negative correlation with M1 macrophages, potentially indicating an inhibitory effect on the anti-tumor immune response within the suppressive microenvironment.

### 3.6. Gene Ontology and Pathway Enrichment Analysis

**Figure 6:**
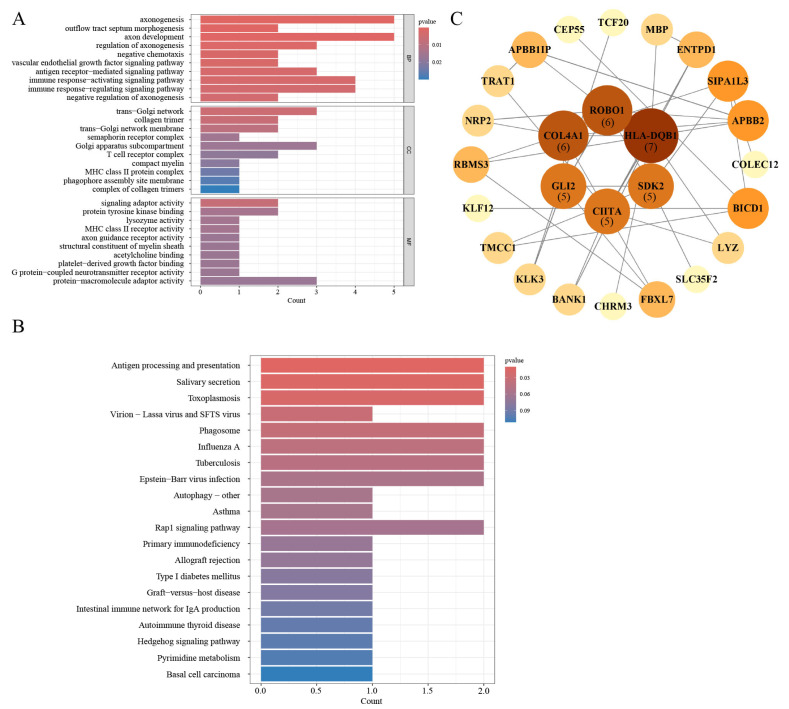
Enrichment analysis and PPI of iDEGs. (**A**) GO enrichment results for three terms of iDEGs. (**B**) KEGG enrichment result of iDEGs. (**C**) PPI network of iDEGs built with STRING. In the PPI network, darker colors and larger spheres represent proteins with more interactions, indicating higher connectivity. Proteins located at the center of the network are labeled with their total connectivity values.

To investigate the functional characteristics and potential biological roles of the iDEGs, enrichment analyses were performed, as detailed in Table S10. As illustrated in Figure 6A, in the Gene Ontology (GO) Biological Process (BP) category, several immune-related processes were significantly enriched, including an immune response-activating signaling pathway, immune response-regulating signaling pathway, and antigen receptor-mediated signaling pathway. In the Cellular Component (CC) category, immune-associated cellular structures such as the MHC class II protein complex and T cell receptor complex were prominently enriched. In terms of Molecular Function (MF), significant enrichment was observed in MHC class II receptor activity, lysozyme activity, and signaling adaptor activity.

Furthermore, enrichment analysis of KEGG pathway reinforced the significant contribution of iDEGs to immune regulation. Specifically, pathways like the “Intestinal immune network for IgA production” were notably enriched, suggesting a potential role for these genes in modulating IgA production and influencing mucosal immunity (Figure 6B). The results indicate that iDEGs are primarily involved in immune-related biological processes and pathways, aligning with the immune-driven characteristics of BC.

### 3.7. PPI Network Construction

To construct the PPI among the iDEGs, we queried the STRING database https://string-db.org/ (accessed on 2 April 2025). This allowed for a comprehensive analysis of potential interactions among the proteins encoded by the identified genes. The resulting interaction data were imported into Cytoscape for visualization. Figure 6C illustrates the network, consisting of 26 nodes and 39 edges, depicting the predicted interactions between the iDEGs and their associated partners (Table S11). Each node represents a protein, while each edge denotes a functional association. Proteins such as HLA-DQB1, ROBO1, and COL4A1 exhibited higher degrees of connectivity, suggesting their central positions within the interaction network.

### 3.8. Selection of Machine Learning Models and Diagnosis Efficacy

**Figure 7:**
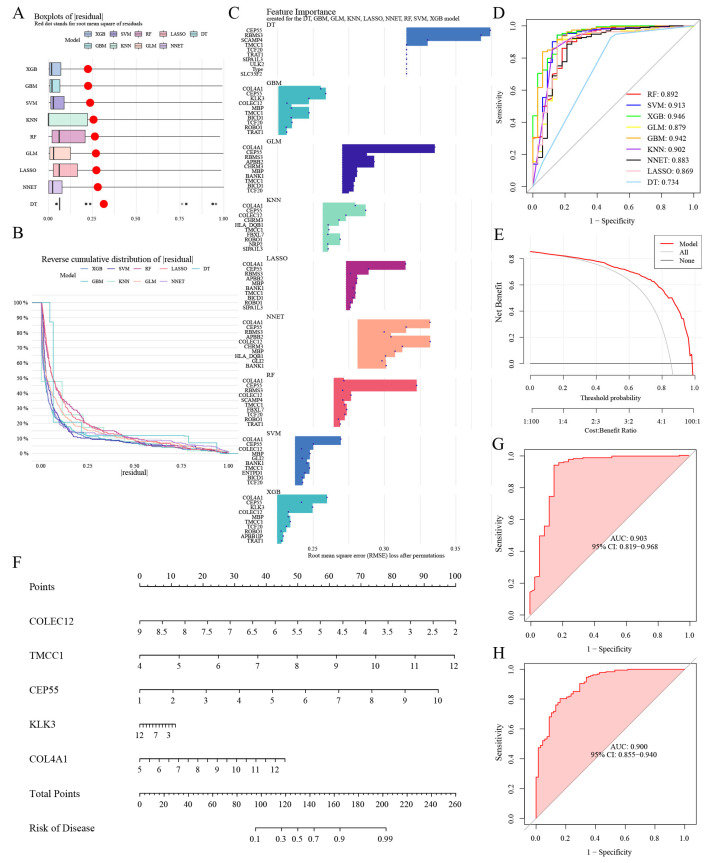
Evaluation of machine learning models and subsequent construction and validation of a nomogram derived from iDEGs. (**A**) Box plots showing the distribution of residuals from the nine machine learning models. Red dots indicate the root mean square of residuals (RMSE), representing the overall prediction error for each model. For the DT model, only residual points are shown due to limited variability, which is insufficient to generate a full boxplot. (**B**) Reverse cumulative distribution of residuals from these models. (**C**) Bar plot showing the top-ranked feature importance scores across nine machine learning models. Each blue dot represents a gene’s permutation-based importance in a given model, based on RMSE increase. Genes without bars were evaluated but not selected among the top features displayed. (**D**) Receiver operating characteristic (ROC) curves for all nine models. (**E**) Decision curve analysis for the nomogram based on feature genes. (**F**) Nomogram developed using the feature genes. (**G**) ROC curve for the combined dataset. (**H**) ROC curve for the GSE13507 validation dataset. The AUC is presented. The machine learning models used include gradient boosting machine (GBM), generalized linear model (GLM), neural network (NNET), k-nearest neighbors (KNN), decision tree (DT), least absolute shrinkage and selection operator (LASSO), random forest (RF), support vector machines (SVM), and extreme gradient boosting (XGB).

Of the nine machine learning models developed, extreme gradient boosting (XGB) demonstrated the highest accuracy, with the lowest residual values (Figure 7A,B) and the greatest area under the receiver operating characteristic curve (Figure 7D). Consequently, this model was selected for further analysis. Finally, feature importance scores were calculated to identify the primary predictors (Figure 7C), with *COLEC12*, *TMCC1*, *CEP55*, *KLK3*, and *COL4A1* emerging as the top five most influential genes (Table S12).

Based on these genes, individual scoring scales were constructed, and the model’s performance was evaluated using the decision curve analysis. The distance between the red and gray lines on the curve served as an indicator of the model’s accuracy (Figure 7E). Furthermore, we calculated the expression scores of these five genes to assess their combined risk in BC (Figure 7F). The predictive power of the model, when using the combined gene sets, resulted in an area under the curve (AUC) value of 0.903 (Figure 7G). Finally, external validation with the GSE13507 dataset confirmed the model’s robustness, yielding an AUC value of 0.900, further demonstrating its excellent predictive accuracy (Figure 7H).

## 4. Discussion

In this study, we conducted an in-depth exploration of the involvement of immune cells in BC by combining MR analysis, differential expression analysis, and machine learning methods. We identified immune cells with potential causal relationships to BC risk, analyzed iDEGs, and developed predictive models using machine learning to identify diagnostic biomarkers.

We have identified eight immune cell types associated with BC risk, involving B cells, T cells, and monocytes. Among these immune cells, the expression of CD19 on transitional B cells, CD20 on IgD− CD24− B cells, CD28 on CD39+ secreting Tregs, HLA-DR+ CD8 bright T cell AC, and IgD on unswitched memory B cells are associated with a reduced risk of BC. In contrast, the expression of HLA-DR on CD14+ CD16− monocytes, HLA-DR on CD14+ CD16+ monocytes, and the percentage of Naive CD4+ T cells are associated with an increased risk of BC.

Current research indicates that B cells play a pivotal role as effector cells within the TME, contributing significantly to the regulation of the anti-tumor immune response [41,42,43]. CD19, a 95 kDa transmembrane glycoprotein, is a crucial biomarker for both normal and tumor-associated B cells, as well as for follicular dendritic cells. It is believed to be instrumental in the activation of B cells [44,45,46]. In the context of MIBC, CD19+ tumor-infiltrating B cells function as APCs, which are essential for activating CD4+ tumor-infiltrating T cells (TIT) [47]. Additionally, the presence of CD19+ B cells has been associated with improved survival outcomes in MIBC patients [47]. On the other hand, CD20, a glycosylated transmembrane phosphoprotein expressed at later stages of B cell activation, serves as a specific marker for mature B cells [48]. Similarly to CD19+ B cells, although the exact role of CD20+ B cells remains unclear, observational studies have shown that an increased presence of CD20+ B cells may serve as a marker for reduced recurrence in non-muscle invasive bladder cancer [49]. CD39+ Tregs are typically known for their immunosuppressive role, facilitating tumor escape from immune surveillance. However, this appears to be in contrast with our findings, where they act as a protective factor in BC. Emerging research indicates that Tregs are not a homogeneous population and may exhibit both pro-tumor and anti-tumor functions, as demonstrated by recent studies on Treg heterogeneity and plasticity, which depend on their microenvironment and the signals they receive [50,51]. The protective effect observed in our study may be associated with the unique phenotype of CD39+ Tregs, which could modulate extracellular adenosine levels in the tumor microenvironment, thereby maintaining immune homeostasis and potentially preventing tumor progression [52,53]. Additionally, further experimental validation is needed to support and clarify this seemingly contradictory result.

HLA-DR is a class II molecule of the major histocompatibility complex (MHC), primarily expressed on antigen-presenting cells (APCs) like dendritic cells and monocytes. CD14+ CD16− monocytes, commonly referred to as classical monocytes, constitute approximately 80–90% of peripheral blood monocytes [54]. These cells predominantly differentiate into tumor-associated macrophages (TAMs), which contribute to the suppression of T cell activity, the recruitment of Tregs, and the promotion of tumor metastasis. These effects are mediated through key signaling pathways, such as NF-κB and STAT3 [55]. Naive CD4+ T cells play a crucial role in shaping tumor immune responses. Upon activation in the TME, these cells differentiate into effector T cells, such as Th1 cells, which enhance the anti-tumor immune response [56,57]. The high proportion of naive CD4+ T cells suggests that the immune system is not effectively activated, enabling bladder cancer cells to evade immune surveillance and promote cancer development, consistent with HLA-DR+ CD8 bright T cells acting as a protective factor. This finding offers valuable insights into the immune mechanisms of BC and potential immunotherapy strategies. Based on the aforementioned studies, our findings are largely consistent with previous research, providing a solid foundation for further investigation.

Using SNPense, we annotated immune cell-related SNPs and performed differential expression analysis, which led to the identification of 28 genes, referred to as iDEGs. Functional enrichment analysis and PPI network construction revealed that these genes are primarily associated with immune functions and related signaling mechanisms. These findings are consistent with the immunogenic characteristics of BC and support the validity of our integrative MR-based differential analysis approach [6,7,8].

In addition, machine learning analysis of the iDEGs identified five key genes—*COLEC12*, *TMCC1*, *CEP55*, *KLK3*, and *COL4A1*—as potential biomarkers for diagnosis. Although current studies on these genes in BC are relatively limited, their reported involvement in other cancer types supports the validity of our findings and suggests that they may represent novel targets for future research. COLEC12 has been shown to regulate apoptosis and inflammatory responses in osteosarcoma, and it promotes enhanced migration and invasion in gastric cancer cells [58,59]. The TMCC family comprises three predicted proteins (TMCC1–3). Although the exact functions and characteristics of these proteins are not yet fully understood, existing studies have suggested that TMCC1 is predominantly localized to the rough endoplasmic reticulum and is involved in critical processes related to endoplasmic reticulum-associated budding [60,61]. *CEP55* has been reported to be overexpressed in BC patients, and research suggests it may promote tumorigenesis by activating the NF-κB signaling pathway [62]. Interestingly, KLK3 (prostate-specific antigen, PSA) is a well-established biomarker in prostate cancer; however, its role in BC remains less well-documented. In our analysis, we suggest that KLK3 may influence the progression of BC. One potential mechanism is extracellular matrix (ECM) remodeling. KLK3, like other kallikrein-related proteases, possesses proteolytic activity that may contribute to ECM component degradation, a critical process for tumor cell invasion and metastasis [63]. Another potential mechanism involves androgen receptor (AR) signaling. *KLK3* expression is regulated by androgens in prostate cancer, and androgen receptor signaling is known to be a key driver of both prostate and bladder cancer progression [64,65,66]. Although the role of KLK3 in regulating AR signaling in BC is less well understood, studies suggest that kallikrein-related proteases influence BC progression by modulating protease activity [67]. This suggests that KLK3 deserves further exploration of its potential function [68]. Furthermore, studies on COL4A1 have revealed that COL4A1 produced by BC cells promotes tumor budding and is associated with poor prognosis. Further analysis of COL4A1 levels in the urine of BC patients has led to the suggestion that COL4A1 may serve as a novel diagnostic and prognostic biomarker [69,70].

## 5. Conclusions

This study explores potential causal links between various immune cells and BC, offering important insights into the mechanisms driving BC and suggesting new pathways for diagnosis and prevention. Our findings highlight the complex interplay between immune cells and BC, identify key molecular markers, and provide a robust foundation for future translational research aimed at unraveling the pathogenesis of BC. While our results have been validated, the study does have limitations. Additional experimental studies are necessary to further confirm the identified biomarkers and elucidate the underlying mechanisms. In future studies, in vitro functional assays will be performed, including knockdown or overexpression experiments of key candidate genes in bladder cancer cell lines. This approach will enable the assessment of how these genes influence tumor cell proliferation, apoptosis, cytokine secretion, and immune response modulation, offering a deeper understanding of the molecular mechanisms underlying bladder cancer progression. Additionally, in vivo studies using xenograft mouse models will examine the impact of these genes on tumor growth and immune cell infiltration. These studies will assess how the identified genetic alterations influence tumor development and the tumor microenvironment, with a particular focus on immune cell populations such as T cells and macrophages. Investigating the effects of these genes in vivo will provide insights into their potential therapeutic applications for bladder cancer. Therefore, ongoing research is required to validate these results and explore their applicability to diverse populations.

## Data Availability

Immune-related trait data were obtained from the GWAS Catalog portal https://gwas.mrcieu.ac.uk/ (accessed on 1 July 2024). Outcome GWAS data for bladder cancer was sourced from FinnGen R11 https://www.finngen.fi/en (accessed on 1 July 2024). Transcriptomic data for bladder cancer, including TCGA-BLCA https://portal.gdc.cancer.gov/ (accessed on 1 July 2024), GTEx-bladder https://xena.ucsc.edu/ (accessed on 1 July 2024), GSE3167 https://ftp.ncbi.nlm.nih.gov/geo/series/GSE3nnn/GSE3167/matrix/ (accessed on 1 July 2024), and GSE13507 https://ftp.ncbi.nlm.nih.gov/geo/series/GSE13nnn/GSE13507/matrix/ (accessed on 1 July 2024), were also utilized.

## Supplementary Materials

The following supporting information can be downloaded at: https://www.mdpi.com/article/10.3390/biomedicines13051203/s1, Table S1: Instrument variables for exposure; Table S2: Bladder cancer related immune cells screened by MR; Table S3: Results of MR Egger intercept horizontal pleitropy test; Table S4: Results of heterogeneity test; Table S5: Results of reverse MR for bladder cancer related immunes; Table S6: Characteristics of the four bladder cancer RNA sequencing datasets; Table S7: SNP conversion results for immune cells associated with bladder cancer using SNPense. Table S8: Differential expression result of iDEGs; Table S9: CIBERSORT analysis of bladder cancer reveals immune cell infiltration; Table S10: Enrichment analysis of iDEGs; Table S11: PPI network of iDEGs; Table S12: Results of XGB model construction.

## Abbreviations

The following abbreviations are used in this manuscript:

BC	Bladder Cancer
NMIBC	Non-muscle-invasive Bladder Cancer
MIBC	Muscle-invasive Bladder Cancer
TME	Tumor Microenvironment
MR	Mendelian randomization
SNPs	Single Nucleotide Polymorphisms
rsIDs	Reference SNP IDs
IVs	Instrumental Variables
IVW	Inverse Variance-weighted
TPM	Transcripts Per Million
iDEGs	Immune-related Differentially Expressed Genes
PPI	Protein–protein Interaction
GO	Gene Ontology
KEGG	Kyoto Encyclopedia of Genes and Genomes
GBM	Gradient Boosting Machine
GLM	Generalized Linear Model
NNET	Neural Network
KNN	K-nearest Neighbors
DT	Decision Tree
LASSO	Least Absolute Shrinkage and Selection Operator
RF	Random Forest
SVM	Support Vector Machines
XGB	Extreme Gradient Boosting
RMSE	Root Mean Square of Residuals
AUC	Area Under the Curve
